# Marked Unilateral Breast Enlargement Caused by a Fibroepithelial Lesion Suspicious for Phyllodes Tumor in the Modern Screening Era: A Case Report and Literature Review

**DOI:** 10.7759/cureus.114300

**Published:** 2026-08-10

**Authors:** Alireza Izadian Bidgoli, Romane Joseph, Shabnam Yazdanpanah, Gabriella Gonzalez

**Affiliations:** 1 General Surgery, American University of the Caribbean School of Medicine, Cupecoy, SXM; 2 General Surgery, Joseph Surgery, Miami, USA; 3 General Surgery, Jackson North Medical Center, Miami, USA

**Keywords:** breast surgery, delayed diagnosis, fibroepithelial lesion, giant breast neoplasm, phyllodes tumor

## Abstract

Markedly enlarged fibroepithelial breast lesions have become increasingly uncommon in the modern era of routine breast imaging and earlier diagnostic evaluation. We report the case of a 52-year-old woman who presented with progressive unilateral right breast enlargement and pain after delaying evaluation despite previously abnormal breast imaging. Clinical examination demonstrated marked right breast enlargement without overt inflammatory or infectious changes. Ultrasound-guided core needle biopsy revealed a fibroepithelial lesion with benign phyllodes tumor included in the differential diagnosis; however, definitive histologic classification required excisional pathology. Given the extensive breast involvement and limited feasibility of breast-conserving surgery, the case was reviewed by a multidisciplinary team, and total mastectomy was recommended. At the time of manuscript preparation, the patient was still considering surgical management, and definitive excisional pathology and long-term follow-up were unavailable. This case highlights the uncommon contemporary presentation of a markedly enlarged fibroepithelial breast lesion despite advances in breast screening and emphasizes the diagnostic limitations of core needle biopsy, the importance of multidisciplinary evaluation, and the consequences of delayed medical assessment on subsequent management.

## Introduction

Fibroepithelial lesions of the breast comprise a spectrum of biphasic neoplasms ranging from fibroadenomas to phyllodes tumors, and distinguishing between these entities can be challenging because of overlapping clinical, radiologic, and histopathologic features, particularly on core needle biopsy [1,2]. Although phyllodes tumors account for less than 1% of all breast neoplasms, they remain clinically important because they may exhibit rapid growth, recur locally, and occasionally demonstrate malignant behavior [1,3]. Contemporary breast screening programs and increased public awareness have led to earlier detection of breast lesions, making markedly enlarged fibroepithelial tumors an increasingly uncommon presentation [2,4].

The diagnosis of phyllodes tumor frequently requires correlation of clinical findings, imaging, and histopathologic evaluation, with definitive classification often relying on examination of the entire excised lesion rather than limited core biopsy specimens [2,5]. Consequently, large or rapidly enlarging fibroepithelial lesions require timely multidisciplinary assessment to guide appropriate surgical management and optimize patient outcomes [3,5].

We report this case to highlight the diagnostic and management challenges of an enlarged fibroepithelial breast lesion suspicious for phyllodes tumor in the modern screening era, emphasizing the impact of delayed presentation on treatment planning and surgical decision-making.

## Case presentation

Clinical presentation

A 52-year-old woman presented to the surgical clinic with progressive right breast swelling associated with intermittent sharp and burning pain that had been worsening over approximately 2.5 months. The patient reported that the pain intensified around her menstrual cycle and was associated with a sensation of warmth over the affected breast. She attempted conservative management with ice packs and warm compresses, which provided only mild symptomatic relief. She denied fever, chills, drainage, nipple discharge, weight loss, erythema, or constitutional symptoms. Her past medical history was notable for alcoholic pancreatitis several years earlier, and a prior benign right breast biopsy performed approximately 22 years before presentation.

The patient had undergone a bilateral screening mammogram approximately one year prior to presentation, which demonstrated multiple oval equal-density masses with circumscribed margins in the right breast central to the nipple at the anterior depth. Additionally, architectural distortion with an indistinct margin was identified in the left breast at the 1 o’clock position. Further diagnostic imaging and ultrasound evaluation had been recommended at that time. The patient had a significant smoking history, reporting combined tobacco and marijuana use since the age of 14 years. 

Initial assessment and physical examination

On presentation, the patient appeared well and was not in acute distress. General physical examination was otherwise unremarkable. Cardiopulmonary and abdominal examinations demonstrated no abnormalities. Neurological examination revealed no gross sensory or motor deficits.

**Figure 1:**
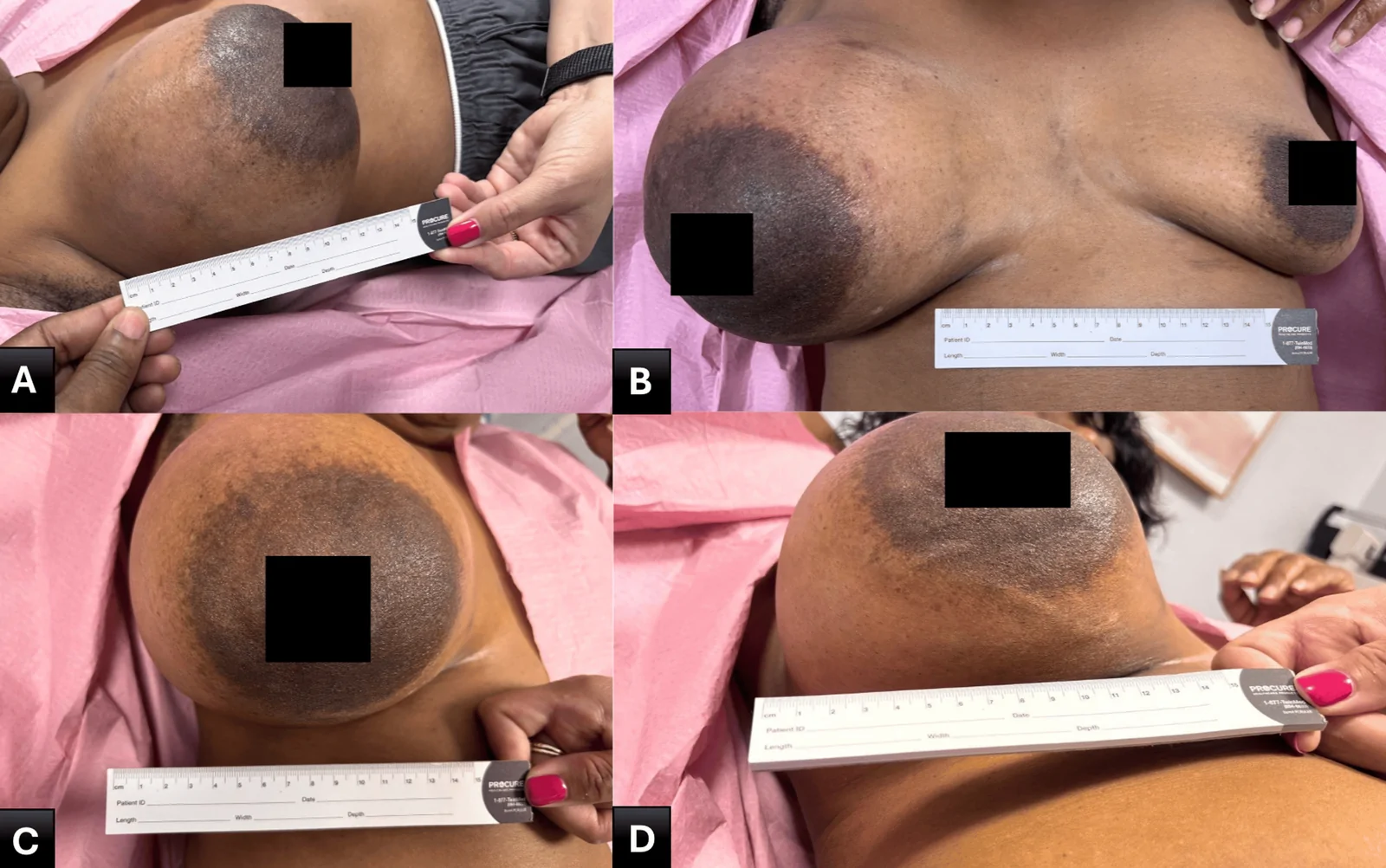
Clinical photographs demonstrating marked unilateral enlargement of the right breast at presentation. (A) Oblique frontal view showing substantial breast enlargement with a measuring ruler for scale. (B) Frontal view demonstrating pronounced asymmetry between the right and left breasts. (C) Superior view highlighting the degree of breast projection and enlargement. (D) Lateral-oblique view demonstrating significant anterior protrusion of the right breast. Despite the extensive breast enlargement, there was no visible skin ulceration, nipple discharge, or overt inflammatory changes. These findings contributed to concern for an underlying fibroepithelial tumor.

Breast examination revealed marked enlargement and swelling of the right breast without overlying skin erythema, skin retraction, nipple retraction, or nipple discharge (Figure 1). The degree of unilateral breast enlargement was striking, resulting in significant asymmetry compared with the contralateral breast. Despite the substantial increase in breast volume, there were no overt signs of skin breakdown, ulceration, or infection. The breast was non-tender on examination, and small palpable lymph nodes were appreciated within the right axilla. No abnormalities were identified in the left breast. Although significant enlargement was present, no distinct palpable mass could be clearly delineated on examination. The clinical impression at that time included mastodynia and an unspecified right breast mass.

Diagnostic testing

**Figure 2:**
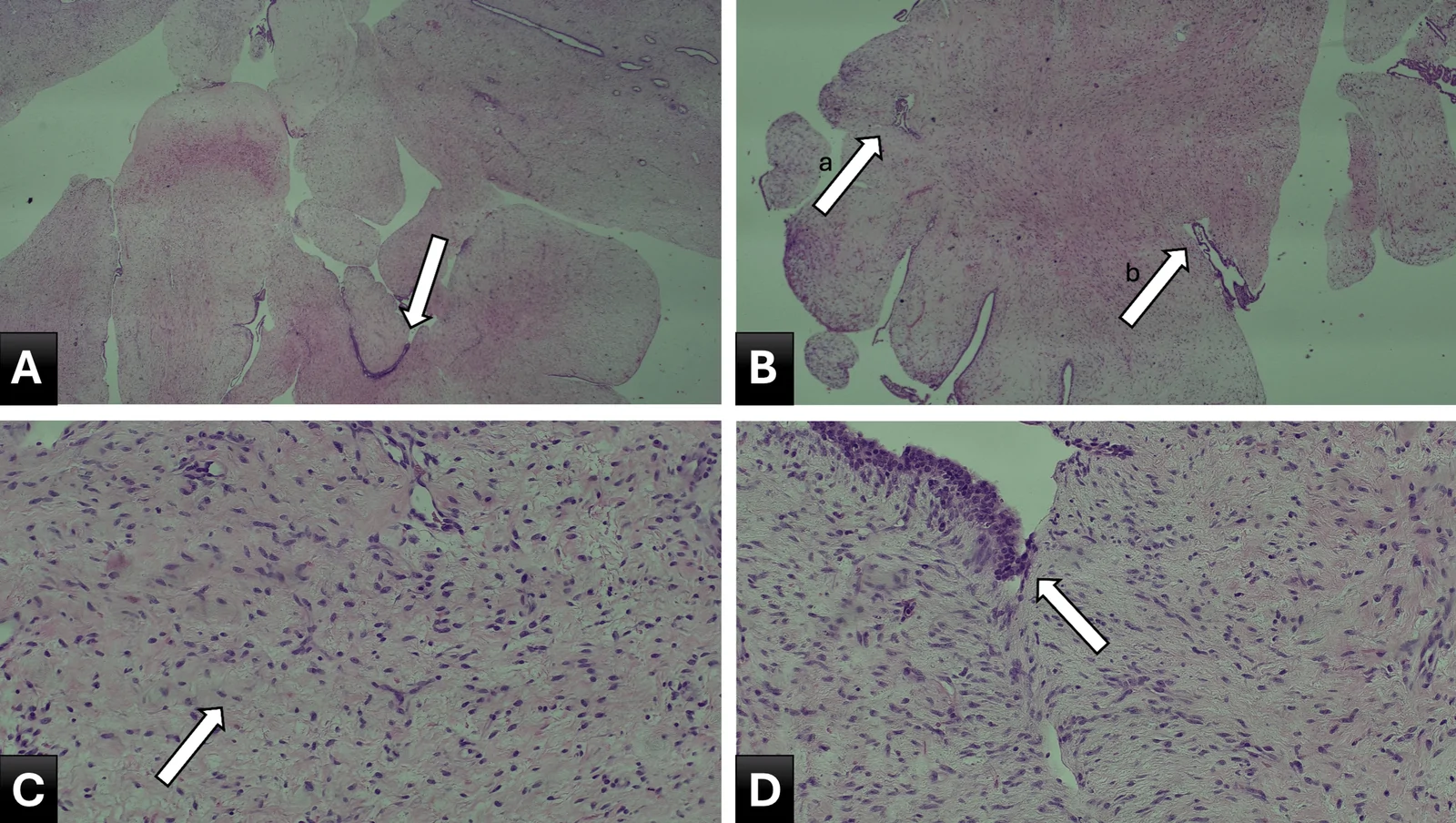
Representative hematoxylin and eosin (H&E)-stained sections from the ultrasound-guided core needle biopsy of the right breast. (A, H&E, ×40) Low-power photomicrograph demonstrating multiple biopsy core fragments containing a biphasic fibroepithelial lesion; the arrow indicates an epithelial-lined cleft. (B, H&E, ×100) Intermediate-power photomicrograph demonstrating the biphasic architecture of the lesion. The left arrow "a" identifies an epithelial-lined cleft, whereas the right arrow "b" highlights the proliferating spindle-cell stromal component. (C, H&E, ×200) High-power photomicrograph demonstrating bland spindle-cell stromal proliferation with mild to moderate cellularity, minimal cytologic atypia, and collagenized stroma. (D, H&E, ×200) High-power photomicrograph demonstrating the epithelial-stromal interface with focal leaf-like architecture, highlighted by the arrow, characteristic of a fibroepithelial lesion. Collectively, these histopathologic findings supported the diagnosis of a fibroepithelial lesion, with benign phyllodes tumor included in the differential diagnosis. Definitive histologic classification was deferred pending excisional biopsy.

Given the patient's progressive unilateral breast enlargement and persistent symptoms, an ultrasound-guided vacuum-assisted core needle biopsy of the right breast was performed with clip placement. Gross examination demonstrated multiple irregular fragments of light tan soft tissue measuring 2.3 × 2.0 × 0.4 cm in aggregate. Histopathologic examination revealed a biphasic fibroepithelial lesion composed of proliferating spindle-cell stroma surrounding epithelial-lined clefts with focal leaf-like architecture (Figure 2). Higher-power examination demonstrated mild to moderate stromal cellularity with bland spindle cells, minimal cytologic atypia, and collagenized stroma without conspicuous mitotic activity in the representative fields (Figure 2). These morphologic findings were consistent with a fibroepithelial lesion, and benign phyllodes tumor was included in the differential diagnosis. However, because of the recognized histologic overlap between fibroadenomas and phyllodes tumors on core needle biopsy, definitive histologic classification was deferred pending excisional biopsy. 

Multidisciplinary board discussion

Following review of the pathology findings, the patient’s case was discussed in a multidisciplinary setting involving the surgical team and medical oncology. The biopsy findings of a fibroepithelial lesion with benign phyllodes tumor included in the differential diagnosis prompted multidisciplinary discussion regarding further diagnostic evaluation and surgical management. Given the significant breast enlargement, persistent swelling, and concern for progression, the surgical team recommended prompt oncologic evaluation and discussed the potential need for definitive surgical resection.

The multidisciplinary consensus favored surgical management because core needle biopsy findings alone were insufficient to definitively classify the fibroepithelial lesion or exclude a phyllodes tumor on core needle biopsy. Due to the extent of breast involvement and clinical presentation, right total mastectomy was discussed as the preferred surgical option.

Diagnosis and management

The patient was diagnosed with a right breast fibroepithelial lesion. Based on the core needle biopsy, benign phyllodes tumor was included in the differential diagnosis; however, definitive histologic classification was deferred pending excisional biopsy. At follow-up evaluation after biopsy, the patient reported overall improvement and denied systemic symptoms or signs suggestive of infection. She described only intermittent pinching discomfort at the biopsy site. Physical examination demonstrated appropriate healing of the biopsy site without erythema, drainage, or evidence of infection.

Management options, including definitive surgical excision for diagnostic and therapeutic purposes, were reviewed extensively with the patient. Given the extensive breast involvement and the inability to definitively classify the lesion on core biopsy, right total mastectomy was recommended after multidisciplinary discussion. The patient elected to defer surgical intervention while considering her treatment options.

Clinical follow-up

At the most recent documented follow-up, the patient remained clinically stable following ultrasound-guided core needle biopsy without evidence of post-procedural complications or infection. The biopsy site demonstrated appropriate healing, and her breast pain had improved, with only intermittent discomfort remaining. The pathology findings and multidisciplinary recommendation for definitive surgical excision were reviewed with the patient. At the time of manuscript preparation, the patient remained undecided regarding surgical management and wished to take additional time before proceeding with treatment. Consequently, definitive excisional pathology and long-term clinical follow-up were unavailable.

## Discussion

Background (history, epidemiology, and risk factors)

Phyllodes tumors are uncommon fibroepithelial neoplasms of the breast that were first described by Johannes Müller in 1838 as “cystosarcoma phyllodes” because of their characteristic leaf-like histologic architecture [5]. Although the term “cystosarcoma” historically implied malignant potential, the World Health Organization (WHO) now classifies phyllodes tumors into benign, borderline, and malignant categories based on stromal cellular atypia, mitotic activity, stromal overgrowth, tumor borders, and necrosis [6].

These tumors account for less than 1% of all breast neoplasms and approximately 2-3% of fibroepithelial breast lesions [6,7]. Phyllodes tumors most commonly occur in women between 40 and 50 years of age, which is approximately one to two decades older than the typical age of presentation for fibroadenomas [6,7]. They usually present as rapidly enlarging unilateral palpable breast masses and are most frequently located within the upper outer quadrant of the breast, although they may arise in any breast region [7].

The etiology of phyllodes tumors remains incompletely understood. However, recurrent MED12 mutations and alterations involving the TERT promoter have been implicated in tumorigenesis [8]. Additional risk factors include prior fibroadenoma, Li-Fraumeni syndrome, and delayed medical evaluation of enlarging breast masses [6,8]. While benign phyllodes tumors generally demonstrate favorable outcomes after surgical excision, borderline and malignant lesions are associated with increased risks of local recurrence and distant metastasis [6,8]. Large or “giant” phyllodes tumors, commonly defined as lesions exceeding 10 cm, are now rarely encountered due to advances in breast imaging and earlier clinical detection [6-8].

Pathogenesis and pathophysiology

Phyllodes tumors are fibroepithelial breast neoplasms that arise from the periductal stromal connective tissue and are characterized by biphasic proliferation of both stromal and epithelial components [5]. Current evidence suggests that the neoplastic process is primarily driven by the stromal component, while the epithelial component remains reactive and non-neoplastic [5]. Histologically, these tumors demonstrate the classic “leaf-like” architecture formed by stromal overgrowth projecting into epithelial-lined clefts [9].

Recent molecular studies have identified recurrent mutations in the MED12 gene as one of the most common genetic alterations in benign and borderline phyllodes tumors, supporting a shared molecular relationship with fibroadenomas [5,6]. Additional alterations involving the TERT promoter, EGFR amplification, TP53 mutations, and chromosomal instability have been associated with progression toward malignant phenotypes and increased risk of recurrence or metastasis [6,9]. Tumor enlargement is largely driven by accelerated stromal proliferation, which can lead to rapid unilateral breast expansion, compression of surrounding tissue, pain, skin stretching, and local deformity, particularly in giant tumors [9].

Unlike conventional breast carcinomas, lymphatic spread is uncommon in phyllodes tumors because metastatic behavior, when present, typically occurs through hematogenous dissemination [9]. The progressive enlargement observed in neglected cases may result in extensive local tissue distortion and limit breast-conserving surgical options, often necessitating mastectomy for adequate local control.

Comparative analysis/literature review 

Clinical Presentation

Large fibroepithelial breast lesions with benign phyllodes tumor included in the differential diagnosis typically present as rapidly enlarging unilateral breast masses in women between 40 and 50 years of age, with progressive enlargement developing over weeks to months [5,10]. Our patient's age and presentation with progressive unilateral breast enlargement were therefore consistent with the demographic and clinical patterns described in the literature [6]. However, the most distinctive feature of this case was the marked extent of breast enlargement despite the modern era of routine mammographic screening and improved access to breast imaging [9,10]. Contemporary screening practices and increased breast health awareness have made markedly enlarged fibroepithelial breast lesions an increasingly uncommon clinical presentation, as most breast abnormalities are evaluated earlier in their course [9,10].

Many reported giant phyllodes tumors present with ulceration, fungation, skin necrosis, or overt inflammatory changes resulting from extensive local expansion [11]. In contrast, our patient exhibited marked unilateral breast enlargement without severe skin compromise, nipple discharge, or overt infection despite prolonged lesion growth [11]. Delayed medical evaluation following previously abnormal breast imaging likely contributed to the advanced clinical presentation observed in this case [9,11]. Although definitive histologic classification was unavailable because excisional pathology had not yet been performed, this case illustrates how delayed evaluation of a progressively enlarging fibroepithelial breast lesion can result in an unusually advanced presentation, even in the contemporary screening era [9,11].

Diagnosis

Radiologically, fibroepithelial lesions suspicious for phyllodes tumor often appear as circumscribed or lobulated masses on mammography and ultrasound, frequently resembling fibroadenomas or other benign fibroepithelial lesions [5,12]. Similar to previously reported cases, our patient's mammogram demonstrated multiple oval equal-density masses with circumscribed margins, findings that lack specificity for phyllodes tumor [12]. The absence of pathognomonic imaging features continues to create substantial diagnostic difficulty in distinguishing phyllodes tumors from fibroadenomas before surgery [5,12].

Histopathologic examination of the core needle biopsy in our patient demonstrated a fibroepithelial lesion, with benign phyllodes tumor included in the differential diagnosis. This closely mirrors findings reported throughout the literature, where core needle biopsy frequently fails to definitively distinguish fibroadenomas from phyllodes tumors because of overlapping epithelial and stromal features [5,10]. Definitive classification generally requires excisional pathology to permit comprehensive assessment of stromal cellularity, cytologic atypia, mitotic activity, stromal overgrowth, tumor borders, and surgical margins [5,12]. In the present case, the absence of excisional pathology precluded definitive histologic classification and limited direct comparison with published series of confirmed phyllodes tumors. Nevertheless, the diagnostic uncertainty encountered in this case reflects a well-recognized limitation of minimally invasive sampling techniques for fibroepithelial breast lesions and highlights the importance of correlating clinical, radiologic, and pathologic findings when planning management [5,10,12].

Management

Complete surgical excision with negative margins remains the cornerstone of management for confirmed phyllodes tumors, regardless of histologic subtype [10,13]. When technically feasible, breast-conserving surgery is generally recommended, whereas mastectomy may be considered for patients with giant tumors, diffuse breast replacement, recurrent lesions, or when adequate surgical margins cannot be achieved while preserving acceptable cosmetic outcomes [9,10,13].

In the present case, multidisciplinary review resulted in a recommendation for right total mastectomy based on the extensive breast involvement, limited feasibility of breast-conserving surgery, and the need to obtain definitive histopathologic diagnosis through excisional tissue evaluation. The recommendation was made in the setting of a fibroepithelial lesion with benign phyllodes tumor included in the differential diagnosis, rather than a confirmed phyllodes tumor. At the time of manuscript preparation, however, the patient had elected to defer definitive surgical intervention while considering her treatment options. Consequently, definitive histopathologic classification and assessment of surgical outcomes were unavailable. Although direct comparison with published surgical series is therefore limited, this case illustrates how delayed presentation of a progressively enlarging fibroepithelial breast lesion may substantially influence surgical planning and reduce the feasibility of breast-conserving management [9,11].

Follow-up Status and Limitations

Published series indicate that prognosis in phyllodes tumors is primarily influenced by histologic grade, completeness of surgical excision, and negative margin status, with borderline and malignant tumors carrying substantially higher risks of local recurrence and distant metastasis than benign lesions [5,10,13]. However, direct comparison of outcomes between the published literature and the present case is not possible because definitive surgical excision had not been performed at the time of manuscript preparation. At the most recent documented follow-up, the patient had recovered uneventfully from core needle biopsy without evidence of procedural complications and reported improvement in breast pain. She remained undecided regarding the recommended total mastectomy. Consequently, definitive histopathologic classification, surgical margin status, recurrence risk, and long-term oncologic outcome could not be determined. Because most published outcome data are based on patients who have undergone definitive surgical treatment, meaningful comparison of prognosis between our patient and previously reported series is not possible [5,10,13].

Summary of the Literature 

Recent literature consistently emphasizes that markedly enlarged fibroepithelial breast lesions suspicious for phyllodes tumor have become increasingly uncommon in the modern screening era because most breast abnormalities are identified and evaluated at earlier stages [9-11]. Systematic reviews and contemporary case series have also highlighted the substantial diagnostic overlap between fibroadenomas and phyllodes tumors on both imaging and core needle biopsy specimens, underscoring the limitations of preoperative diagnosis [5,12]. Published reports of confirmed giant phyllodes tumors frequently describe advanced presentations with ulceration, hemorrhage, chest wall distortion, or recurrent disease requiring extensive surgical resection [11,13]. In contrast, our patient presented with marked unilateral breast enlargement without severe skin compromise despite prolonged progression and delayed medical evaluation [11].

Because definitive excisional pathology was unavailable, direct comparison with published series of confirmed phyllodes tumors is necessarily limited. Nevertheless, this case highlights the clinical consequences of delayed evaluation following previously abnormal breast imaging and demonstrates how prolonged progression of a large fibroepithelial breast lesion may substantially influence surgical planning [7-9]. The diagnostic uncertainty encountered in this case further reinforces the importance of multidisciplinary assessment, careful clinicopathologic correlation, and timely evaluation of enlarging breast lesions when core needle biopsy identifies a fibroepithelial lesion with phyllodes tumor included in the differential diagnosis [10,12].

**Table 1:** Comparison of the present case with contemporary literature on fibroepithelial breast lesions and phyllodes tumors This table summarizes the clinical presentation, diagnostic evaluation, histopathologic findings, management considerations, and available follow-up of the present case in comparison with patterns reported in the contemporary literature. Because definitive surgical excision had not been performed, comparisons regarding treatment outcomes are limited.

Feature	Typical Findings in Literature	Findings in Our Case	Clinical Significance
Patient Demographics	Most phyllodes tumors occur in women aged 40-50 years [5,10].	52-year-old female	Consistent with the common age distribution reported in the literature
Clinical presentation	Rapidly enlarging unilateral palpable breast mass [5,10].	Progressive unilateral right breast enlargement with burning pain and warmth	Typical symptom pattern but unusually advanced local enlargement
Tumor size	Giant phyllodes tumors (>10 cm) are now uncommon due to earlier detection [9,10].	Marked breast enlargement at presentation	Highlights the consequences of delayed medical evaluation in the modern screening era
Skin changes	Large tumors often present with ulceration, fungation, or skin necrosis [11].	No ulceration, infection, nipple discharge, or skin necrosis	Atypical presentation despite extensive tumor growth
Imaging findings	Circumscribed or lobulated breast masses often resembling fibroadenoma [5,12].	Multiple oval equal-density circumscribed masses on mammography	Reinforces the nonspecific radiologic appearance of phyllodes tumors
Diagnostic challenge	Core needle biopsy frequently unable to definitively distinguish fibroadenoma from phyllodes tumor [5,10].	Fibroepithelial lesion with benign phyllodes tumor included in differential diagnosis	Demonstrates limitations of minimally invasive tissue sampling
Histopathology	Definitive diagnosis often requires excisional pathology to distinguish fibroadenoma from phyllodes tumor [5,12].	Core needle biopsy demonstrated a fibroepithelial lesion with benign phyllodes tumor included in the differential diagnosis; definitive classification was deferred pending excisional pathology	Illustrates the recognized limitations of core needle biopsy for definitive classification of fibroepithelial lesions.
Management	Complete surgical excision is recommended once diagnosis is established, with the extent of surgery based on lesion size and the ability to achieve adequate margins [10,13].	Multidisciplinary recommendation for right total mastectomy; definitive surgery had not been performed at the time of manuscript preparation	Demonstrates how extensive lesion size may influence surgical planning while emphasizing that definitive treatment remained pending
Breast-conserving feasibility	Breast conservation preferred when technically feasible [5].	Not feasible because of extensive breast involvement	Delayed presentation significantly limited surgical options
Metastatic pattern	Malignant phyllodes tumors spread hematogenously rather than lymphatically [9].	Small axillary lymph nodes without confirmed metastatic disease	Consistent with the known biology of phyllodes tumors
Follow-up status	Prognosis and recurrence are determined after definitive surgical excision and depend on histologic grade and surgical margin status [5,10,13].	Short-term recovery after core needle biopsy; definitive surgery and excisional pathology unavailable at the time of manuscript preparation.	Long-term oncologic outcome cannot be determined or directly compared with published postoperative outcomes.
Unique contribution of this case	Giant breast lesions have become increasingly uncommon in the modern screening era because most abnormalities are detected earlier [9-11]	Marked unilateral breast enlargement despite previously abnormal imaging and delayed medical evaluation, with persistent diagnostic uncertainty on core biopsy	Highlights the consequences of delayed follow-up and the diagnostic limitations of core needle biopsy in large fibroepithelial breast lesions

What have we learned from this case?

This case highlights an increasingly uncommon but clinically important presentation of a markedly enlarged fibroepithelial breast lesion in the modern era of widespread breast imaging and screening. The most distinctive aspect of this case was not simply the size of the lesion, but the fact that such advanced local progression occurred despite previously abnormal breast imaging and ready access to diagnostic evaluation. Rather than reflecting a failure of contemporary screening technology, this case illustrates how delayed follow-up of abnormal findings can allow a breast lesion to progress to an extent that is now rarely encountered in routine clinical practice.

An important diagnostic lesson is that persistent unilateral breast enlargement should prompt thorough evaluation, even in the absence of skin ulceration, nipple discharge, constitutional symptoms, or other classic features of locally advanced breast disease. In this patient, progressive enlargement, intermittent pain, and nonspecific imaging findings created a diagnostic challenge that could not be resolved by clinical examination or imaging alone. The inability of core needle biopsy to definitively distinguish between fibroadenoma and phyllodes tumor further emphasizes a well-recognized limitation of minimally invasive tissue sampling for fibroepithelial lesions and reinforces the need for careful clinicopathologic correlation when biopsy findings do not fully explain the clinical presentation.

From a management perspective, this case demonstrates that multidisciplinary evaluation remains essential when imaging, pathology, and clinical findings are discordant or when definitive histologic classification is unavailable. Although the patient had not yet undergone definitive surgical treatment, the extent of breast enlargement substantially influenced surgical planning by limiting the feasibility of breast-conserving management and prompting a recommendation for total mastectomy. Finally, this case underscores the importance of patient education and timely follow-up after abnormal breast imaging. Even in the modern screening era, delayed evaluation may permit marked lesion progression, complicate diagnostic assessment, and reduce available treatment options. The educational value of this report therefore lies not in a confirmed diagnosis, but in illustrating the diagnostic uncertainty, multidisciplinary decision-making, and clinical consequences of delayed presentation in a patient with a large fibroepithelial breast lesion suspicious for phyllodes tumor.

## Conclusions

This case highlights the diagnostic and management challenges of a large fibroepithelial breast lesion with benign phyllodes tumor included in the differential diagnosis in a patient who presented after delayed medical evaluation despite progressive unilateral breast enlargement and previously abnormal breast imaging. The case illustrates the limitations of core needle biopsy in definitively distinguishing fibroepithelial lesions from phyllodes tumors and emphasizes the importance of integrating clinical, radiologic, and pathologic findings through a multidisciplinary approach. It also demonstrates how delayed presentation may substantially influence surgical planning by limiting breast-conserving options, even before a definitive histologic diagnosis is established. A limitation of this report is the absence of excisional pathology and long-term clinical follow-up at the time of manuscript preparation, precluding definitive histologic classification, assessment of surgical outcomes, and evaluation of recurrence risk. Nevertheless, this case reinforces the importance of timely evaluation of progressively enlarging breast masses to facilitate appropriate diagnosis, multidisciplinary decision-making, and optimal patient management.
